# Conformational bifurcation drives dual transport regimes in molecular junctions: Unsupervised machine learning insights

**DOI:** 10.1002/smo2.70033

**Published:** 2026-01-17

**Authors:** Yuhong Chen, Ioan Bâldea, Elad Koren, Zuoti Xie

**Affiliations:** ^1^ Department of Materials Science and Engineering MATEC Guangdong Technion‐Israel Institute of Technology Shantou Guangdong China; ^2^ Department of Materials Science and Engineering Technion‐Israel Institute of Technology Haifa Israel; ^3^ Theoretical Chemistry Heidelberg University Heidelberg Germany; ^4^ Quantum Science Center of Guangdong‐Hong Kong‐Macao Greater Bay Area (Guangdong) Shenzhen‐Hong Kong International Science and Technology Park Shenzhen Guangdong China

**Keywords:** charge transport, conformational bifurcation, molecular electronics, molecular junctions, n‐alk‐1‐ynes, structure‐property relationships

## Abstract

Control over charge transport in molecular–scale devices requires a deep understanding of how minute structural changes influence electronic properties. Here, we demonstrate dual transport regimes in tunnel junctions of *n*‐alk‐1‐yne (CnA) molecules with gold electrodes driven by conformational bifurcation—the emergence of two nearly isoenergetic (planar and skewed) molecular conformers (dihedral angles α=180°andα≈65° at the alkyne terminus in the gas phase). Although the energy differences are small, these subtle conformational differences manifest as distinct transport behaviors, uncovered through unsupervised machine learning, which identified two junction groups: “short” and “long” chains, with distinct attenuation factors ($\beta_{\text{short}}\approx 1.0$ vs. $\beta_{\text{long}}\approx 0.74$) and contact conductances ($G_{c,\text{short}}\approx 200\;\mu S$ vs. $G_{c,\text{long}}\approx 8\;\mu S$). This dramatic impact of the dihedral angle exceeds the impact of the inter‐ring twist angle in biphenyl‐based junctions and rivals changes induced by switching from gold to platinum electrodes or from monothiol to dithiol anchors in oligoacene and oligophenylene junctions. X‐ray photoelectron spectroscopy (XPS) confirmed this bifurcation, linking the “short” and “long” groups to planar and skewed conformers, with dihedrals remarkably agreeing with the gas‐phase values. This work establishes conformational bifurcation as a promising route for designing programmable nanotransport properties through anchor‐group control.

## Graphical abstract

Conformational bifurcation in n‐alk‐1‐yne molecular junctions drives dual transport regimes, with unsupervised machine learning and XPS revealing distinct planar and skewed conformers influencing conductance and tunneling efficiency. This discovery highlights anchor‐group control as a key design principle for tailoring molecular electronic devices.

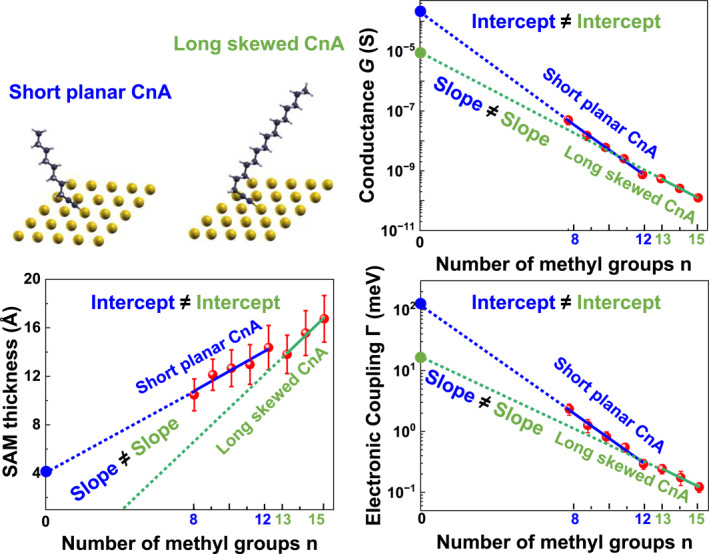

## 1 INTRODUCTION AND BACKGROUND

The quest to harness molecules as functional components in electronic devices hinges on a fundamental challenge: understanding how atomic‐scale structural variations govern charge transport. While the exponential decay of conductance with length in molecular tunnel junctions is a well‐established paradigm in molecular electronics (Equation (1)), the implicit assumptions on which it relies—(i) molecular length scales linearly with repeat units (n), and (ii) all molecules share identical contact properties (including the electronic coupling at the interface)—are routinely overlooked and can obscure subtler structure‐property relationships. Here, we demonstrate that conformational bifurcation—the emergence of two distinct yet energetically comparable molecular conformers—can create dual transport regimes within seemingly uniform molecular junctions, with profound implications for device design.

In conventional semiconductor devices, contact resistances $\left(R_{c}\right)$ and material resistances $\left(R_{m}\right)$ combine additively $\left(R=R_{c}+R_{m}\right)$, with $R_{c}$ becoming negligible at large lengths $\left(R_{c}/R_{m}∼O\left(1/L\right)\right)$. Molecular tunnel junctions defy this intuition: contacts contribute multiplicatively to resistance, and their impact persists regardless of molecular length. This behavior is typically described by the low‐bias conductance formula.[^1^, ^2^, ^3^, ^4^, ^5^, ^6^, ^7^]

(1) $$G=G_{c}\;\exp\left(-\beta n\right)=G_{c}^{L}\;\exp\left(-\beta_{L}L\right)$$

where $G_{c}$ is the contact conductance and β the attenuation factor. Crucially, Equation (1) assumes that (i) molecular length scales linearly with repeat units (n), and (ii) all molecules share identical contact properties (including the electronic coupling at the interface). Violations of these assumptions—such as the conformational bifurcation at anchor groups—can lead to misinterpretations of transport mechanisms. While conformational control of conductance is a recognized principle in molecular electronics—exemplified by the canonical dependence on the inter‐ring twist angle in biphenyl systems—the specific mechanism we report here is fundamentally different. It involves a bifurcation in the *dihedral angle of the terminal anchor group itself* relative to the molecular backbone, a phenomenon distinct from torsions within the π‐system and one that, to the best of our knowledge, has not been previously demonstrated to yield two discrete and stable transport regimes within a single molecular series.

Our investigation focuses on *n*‐alk‐1‐yne (CnA) molecules, where quantum chemical calculations reveal a striking duality: two nearly isoenergetic conformers exist for the terminal alkyne group, significantly differing only in dihedral angle—planar (α=180°) versus skewed (α≈65°) (Supporting Information S1: Figures S1 and S2). While these rotamers are separated by 0.1−0.2 kcal/mol in the gas phase^[8]^—making them thermally interchangeable ($k_{B}T\approx 0.6$ kcal/mol at room temperature)—their adsorption on gold electrodes could preferentially stabilize one conformer, with cascading effects on transport.

**FIGURE 1:**
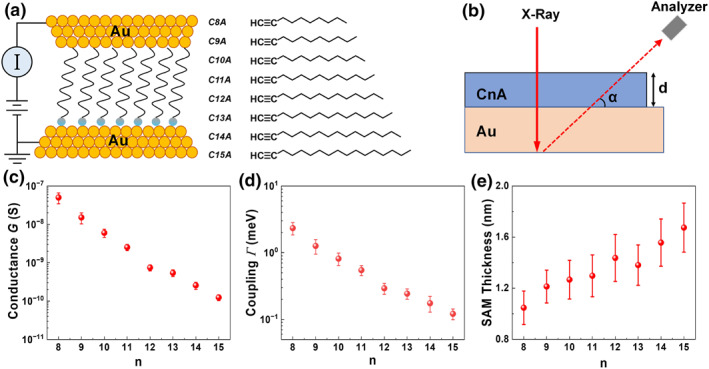
Baseline experimental characterization. (a) Transport measurement schematic for molecular tunnel junctions based on self‐assembled monolayers (SAMs) of n‐alk‐1‐ynes (CnA) with gold electrodes and (b) Angle‐resolved X‐ray photoelectron spectroscopy (XPS) analysis setup. Raw experimental outputs shown for (c) conductance G, (d) MO‐electrode coupling Γ, and (e) thickness measurements d, which do not provide any obvious visual or statistical cues on possible data partitioning.

**FIGURE 2:**
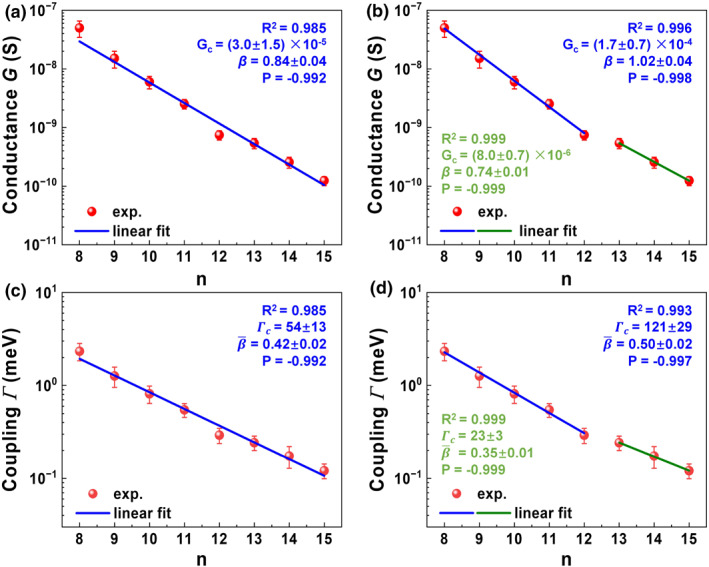
Dual Transport Regimes in CnA Conducting Probe Atomic Force Microscopy Junctions Revealed by Unsupervised Machine Learning. (a) Single exponential fit of ln(G) versus molecular length (n), assuming uniform $G_{c}$ and β (Equation (1)). (b) ML‐identified “short” (n=8–12) and “long” (n=13–15) groups with distinct slopes (ln(G): −1.020 vs. −0.738, z‐score: −7.551, p<0.001). (c, d) Corresponding ln(Γ) analysis (slopes: −0.499 vs. −0.348, z‐score: −5.869, p<0.001). Two‐group fits yield high goodness‐of‐fit ($R^{2}>0.993$, Pearson correlation |P|>0.996) compared to the single‐group model. Significant differences in slopes and means (Welch's *t*‐test, ln(G): t=3.772, p=0.010; ln(Γ): t=3.846, p=0.009) confirm distinct transport regimes. A silhouette score of 0.388 and bootstrap stability (76.2%–81.2%) validate robust clustering.

Unsupervised machine learning (ML) provides an ideal tool to probe this hypothesis. Unlike supervised methods requiring pre‐labeled data, unsupervised ML identifies latent patterns directly from measurements. For CnA junctions fabricated and measured via Conducting Probe Atomic Force Microscopy (CP‐AFM) (Figure 1a), straightforward analysis of conductance G, effective molecular orbital‐electrode (MO‐electrode coupling) Γ—mathematically defined as the geometric average (Supporting Information S1: Equation (S1b)) of the electronic couplings of the dominant molecular orbital (HOMO‐1 in the case of CnA^[9]^) to the substrate $\left(\Gamma_{s}\right)$ and tip $\left(\Gamma_{t}\right)$ electrodes—and self‐assembled monolayer (SAM) thickness d offered no immediate visual cues for data bipartitioning (Figure 1c–e). Yet, as we demonstrate, ML algorithms robustly partition the data into two groups—“short” (n=8–12) and “long” (n=13–15) chains—with statistically distinct β and $G_{c}$ values (Figure 2).

**FIGURE 3:**
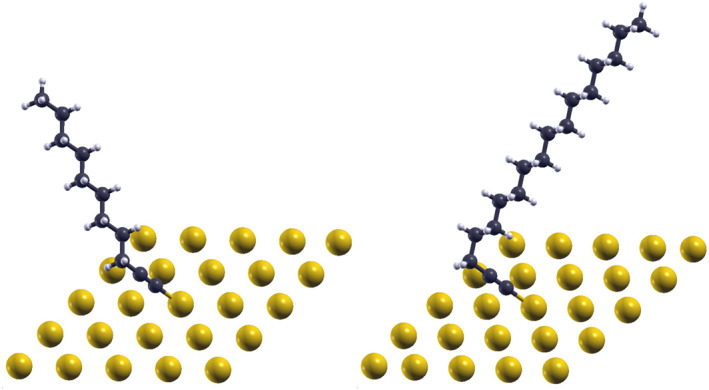
Conformational Differences in “Short” and “Long” CnA Self‐Assembled Monolayers (SAMs). The figure illustrates the distinct orientations and internal conformations of “short” (C9A, left) and “long” (C15A, right) CnA species on a gold substrate. The internal conformation is quantified by the dihedral angle α, which was determined from the dual group fitting analysis (Section 2.5). The measured dihedral angles for the “short” (α≈176°) and “long” (α≈65°) conformers closely match the α values obtained from quantum chemical calculations for the gas‐phase $C_{s}$ planar (α=180°) and $C_{1}$ skewed (α=65°) conformations, respectively. See also Supporting Information S1: Figures S7 and S8 for depictions of all short and long species investigated. Figure generated using XCrySDen.^[10]^

Companion angle‐resolved X‐ray photoelectron spectroscopy (XPS) (Figure 1b) confirms that these groups correspond to planar and skewed conformers, respectively, with dihedral angles matching gas‐phase predictions (dihedral angles 176° vs. 65°; Figure 3 and Supporting Information S1: Figures S7 and S8). The aforementioned dihedral‐angle‐driven bifurcation produces stark differences in $G_{c}$ (200μS vs. 8μS) and β (1.0 vs. 0.74), comparable to switching electrode materials (Au to Pt) or anchor groups (monothiol to dithiol) in oligoacene^[11]^ and oligophenylene systems,[^12^, ^13^] offering a new lever for nanoscale device design.

This work establishes conformational bifurcation as both a hidden variable and mechanistic origin of dual transport regimes in molecular electronics, enabling deliberate control of transport regimes through the internal conformation of the anchor‐group geometry. The synergy of ML with experimental and theoretical tools resolves a structure‐property relationship invisible to conventional approaches. By integrating quantum chemistry, transport measurements, XPS, and unsupervised ML, we provide a template for uncovering latent structure‐property relationships in other nanoscale systems.

The following sections detail our experimental and theoretical methodology (Section 4.1), present the bifurcation's electronic and structural signatures (Section 2), and discuss implications for molecular device engineering (Section 3).

## 2 RESULTS AND DISCUSSION

### 2.1 Theoretical background: Conformational landscape of n‐alk‐1‐ynes—Planar versus skewed rotamers

Our interest in the molecular conformation of *n*‐alk‐1‐ynes (CnA) was sparked by a notable inconsistency: structures from common sources (ChemBio3D, NIST,^[14]^ PubChem,^[15]^ and ChemSpider^[16]^) show significant discrepancies. This observation prompted our joint experimental and theoretical investigation into CnA molecular conformations, focusing on the orientation of the terminal alkyne relative to the planar backbone of the alkyl chain.

Quantum chemical calculations, using state‐of‐the‐art compound model thermochemistries (CBS‐QB3, CBS‐4M, G4) in Gaussian 16,^[17]^ revealed a nuanced conformational landscape for isolated CnA molecules (more details make the object of a separate theoretical publication^[8]^). It consists of two nearly degenerate rotamers (energy difference ≈0.1−0.2 kcal/mol): a planar conformer ($C_{s}$ point group) and a skewed (nonplanar) conformer ($C_{1}$ point group). In the planar conformer, the terminal alkyne is coplanar with the alkyl backbone, while in the skewed conformer, it is rotated out‐of‐plane.

The dihedral angle at the alkyne terminus—defined by carbon atoms C2, C3, C4, C5 in IUPAC notation (starting from the triple bond $\text{–}\;C_{1}\equiv C_{2}\text{–}\;$) (Supporting Information S1: Figure S2)—is 180° for the $C_{s}$ conformer and ∼65° for the $C_{1}$ conformer. The $C_{1}$ conformer closely resembles the ideal “gauche” conformation (typically 60° in alkanes), indicating a slightly relaxed staggered arrangement compared to pure alkane chains.

The near‐degeneracy of these conformers arises from a subtle interplay of electronic and steric interactions. The torsional strain from the dihedral collapse from 180° (planar $C_{s}$) to ≈65° (skewed $C_{1}$) is nearly balanced by the relief of steric repulsions, potentially enhanced by long‐range dispersion interactions. This fine balance yields a potential energy surface with two accessible low‐energy minima. The small energy difference (0.1−0.2 kcal/mol^[8]^)—albeit well below the standard “chemical accuracy” (∼1 kcal/mol) that the most accurate computational methods currently available strive for even for molecules of smaller size[^18^, ^19^, ^20^] is also well below thermal energy at room temperature ($k_{B}T\approx 0.6$ kcal/mol)—suggests that both rotamers are relevant for transport data analysis. Whether environmental constraints in a SAM preferentially stabilize one conformer in a length‐dependent manner is an open question that our subsequent data analysis objectively addresses, without presupposing such an outcome.

In closely packed SAMs on metal electrodes, however, this gas‐phase picture may not hold. Environmental constraints, such as tight packing and adsorption forces, may preferentially stabilize one conformer, potentially influenced by molecular length. Such selective stabilization could significantly affect electronic transport properties, which are highly sensitive to the electronic coupling at the interface, dictated by the internal conformation of the anchor group and molecular topology. In biphenyl‐based junctions, for example, the inter‐ring twist angle δ is known to affect the conductance.[^1^, ^21^, ^22^, ^23^]

(2) $$G\propto \cos^{2}\;\delta \overset{\delta =180{}^{\circ}\to 65{}^{\circ}}{\to }G_{c}^{\mathrm{skewed}}=0.19\;G_{c}^{\mathrm{planar}}\approx G_{c}^{\mathrm{planar}}/5$$

If the cosine‐square scaling (Equation (2)) held for CnA, the contact conductance of the two rotamers would differ by a factor of five, prompting our investigation of electronic transport to probe these effects.

### 2.2 Transport properties

**FIGURE 4:**
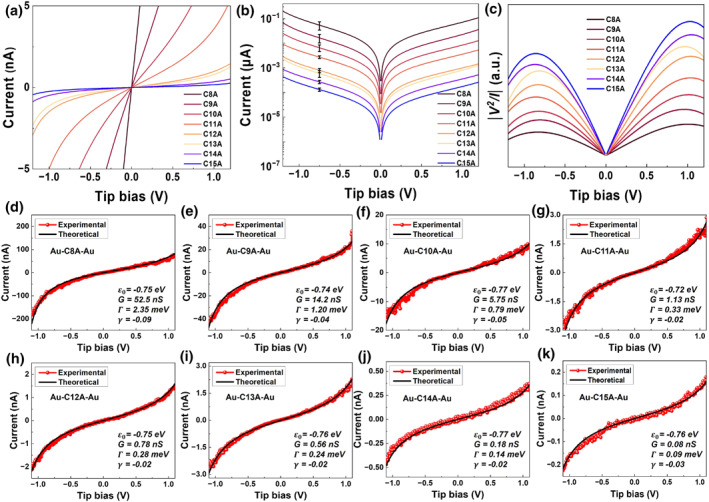
Transport Properties of Molecular Junctions of CnA and Gold Electrodes. (a) Average current‐voltage (I‐V) characteristics, (b) semilogarithmic replot, and (c) $V^{2}/|I|$ versus V representation (whose peaks define the transition voltages $V_{t\pm }$, Supporting Information S1: Equation (S2). (d–k) Representative raw I‐V traces for C8A–C15A junctions with theoretical fits using the off‐resonant single‐level model (Supporting Information S1: Equation (S1a)).

The analysis of the conductance G presented below is model independent; G can be directly extracted from the slope of the I–V curves measured at low bias. To gain more microscopical insight we have also examined the effective MO‐electrode coupling Γ. To this aim, we used the off‐resonant single level model (more details in the Supporting Information S1).^[24]^ Although a more general single level model became available recently,[^25^, ^26^] this model suffices to accurately reproduce the measured I–V curves in the whole bias range investigated (Figure 4d–l). It allows to determine the values of the energy offset $\varepsilon_{0}$($=-|\varepsilon_{0}|<0$) of the dominant molecular orbital (which is the HOMO‐1 in the case of CnA junctions^[9]^), which turned out to agree well with estimates obtained by corroborating data of ultraviolet photoelectron spectroscopy (UPS) and ab initio quantum chemical calculations.^[9]^ The practically negligible dependence on n of the level energy offset $\varepsilon_{0}$ is due to the localized (HOMO‐1^[9]^) on the acetylene group.

With the values of G and $\varepsilon_{0}$ derived as described above we were able to estimate the effective MO‐electrode coupling Γ (Supporting Information S1: Equation (S1c))

(3) $$\Gamma =\Gamma_{c}^{L}\;\exp\left(-{\bar{\beta }}_{L}L\right)=\Gamma_{c}\;\exp\left(-\bar{\beta }n\right)$$

All values of G and Γ needed for the analysis that follows are collected in Table 1.

**TABLE 1:** Key experimental results for CnA CP‐AFM junctions with gold electrodes.

n	Conductance G (nS)	$\left|\varepsilon_{0}\right|$ (eV)	Γ (meV)	Thickness (Å)
8	50 ± 16	0.79 ± 0.02	2.33 ± 0.49	10.5 ± 1.3
9	15 ± 4.8	0.78 ± 0.03	1.26 ± 0.31	12.1 ± 1.3
10	6.0 ± 1.5	0.79 ± 0.03	0.81 ± 0.17	12.7 ± 1.5
11	2.5 ± 0.5	0.81 ± 0.02	0.55 ± 0.09	13.0 ± 1.6
12	0.75 ± 0.13	0.80 ± 0.02	0.29 ± 0.05	14.4 ± 1.8
13	0.54 ± 0.11	0.78 ± 0.04	0.24 ± 0.04	13.8 ± 1.6
14	0.26 ± 0.06	0.82 ± 0.04	0.17 ± 0.05	15.6 ± 1.8
15	0.12 ± 0.02	0.81 ± 0.04	0.12 ± 0.02	16.7 ± 1.9

*Note*: SAM thickness, low‐bias conductance G, MO energy offset $\varepsilon_{0}$, and effective MO‐electrode coupling Γ.

### 2.3 Machine learning analysis of the transport properties

Direct inspection of the conductance (G) and molecule‐electrode coupling (Γ) data in Table 1 and Figure 1c,d reveals no obvious visual or statistical evidence of distinct transport regimes among CnA junctions. However, the conformational bifurcation identified in Section 2.1 suggests that CnA junctions may form two groups—one with planar conformers and another with skewed conformers—potentially influencing transport properties. This hypothesis naturally frames a bipartition problem, aiming to correlate molecular structure with distinct transport or geometric behaviors.

The combinatorial complexity of evaluating possible groupings underscores the need for an unsupervised ML approach. For eight molecular lengths (N→8, C8A to C15A), the number of possible bipartitions is given by the Stirling number of the second kind, $S\left(N,2\right)=2^{N-1}-1\to 127$. Manually testing all 127 partitions via fitting or hypothesis testing is impractical without prior rationale.

Unsupervised ML, which identifies patterns without predefined labels, is ideal for uncovering hidden subpopulations in the absence of clear cues from raw data (Figure 1c,d). Such data‐driven approaches are increasingly valuable for extracting latent knowledge from complex experimental datasets, a principle that is also being applied to map the evolution of entire scientific fields.^[27]^

We applied unsupervised ML to cluster the logarithmic values of conductance (ln(G)) and MO‐electrode electronic coupling (ln(Γ)) from Table 1, measured as a function of molecular size (n). The core advantage of ML here is its ability to objectively identify the optimal bipartition. The conformational bifurcation manifests not as distinct clusters in a single parameter but as two superimposed linear trends in the lnG versus n and lnΓ versus n relationships. ML efficiently evaluates all 127 partitions to find the one that yields the two most statistically distinct and internally consistent groups, a task that is infeasible to perform objectively by hand. Note that G is derived model‐independently from I–V curves, while Γ is calculated using the off‐resonant single‐level model of transport (Supporting Information S1: Section S2) [24]. A synthetic dataset mimicking the real data was used for validation (Supporting Information S1: Section S4). To make the approach accessible to molecular electronics researchers unfamiliar with ML, we draw below analogies to chemical concepts:

Hierarchical clustering: Hierarchical clustering organizes data hierarchically, merging points (junction's ln(G) and ln(Γ)) based on similarity, akin to grouping molecules by structural features.^[28]^ Using Ward's linkage to minimize within‐cluster variance, we identified two groups with a silhouette score of 0.388, indicating significant separation.^[29]^

K‐means clustering: K‐means clustering partitions data into two groups by optimizing their centroids, similar to classifying molecules into distinct reaction types based on properties.^[30]^ After 10 initializations, it yielded identical groups to hierarchical clustering (silhouette score 0.388, adjusted Rand index 1.000).^[31]^

Gaussian mixture models (GMM): GMM assumes data arise from two overlapping distributions, analogous to molecules exhibiting distinct behaviors in a mixture.^[32]^ For the small dataset in Table 1, GMM converged to the same groups as hierarchical and K‐means clustering (silhouette score 0.388, adjusted Rand index 1.000).

Further details are provided in the Supporting Information S1. Detailed ML numerical results are summarized in Table 2.

**TABLE 2:** Clustering results for CnA transport data.

Metric	Hierarchical	k‐means	GMM
Silhouette score	0.388	0.388	0.388
Davies‐Bouldin index	0.724	0.724	0.724
Adjusted Rand index	–	1.000	1.000
Cluster “short” (n)	[8–12]	[8–12]	[8–12]
Cluster “long” (n)	[13–15]	[13–15]	[13–15]
Conductance slope (cluster “short”)	−1.020	−1.020	−1.020
Conductance $R^{2}$ (cluster “short”)	0.996	0.996	0.996
Gamma slope (cluster “short”)	−0.499	−0.499	−0.499
Gamma $R^{2}$ (cluster “short”)	0.993	0.993	0.993
Conductance slope (cluster “long”)	−0.738	−0.738	−0.738
Conductance $R^{2}$ (cluster “long”)	1.000	1.000	1.000
Gamma slope (cluster “long”)	−0.348	−0.348	−0.348
Gamma $R^{2}$ (cluster “long”)	0.999	0.999	0.999
Conductance slope z‐score	−7.551	−7.551	−7.551
Conductance slope z‐score *p*‐value	0.000	0.000	0.000
Gamma slope z‐score	−5.869	−5.869	−5.869
Gamma slope z‐score *p*‐value	0.000	0.000	0.000
Conductance Normality *p*‐value (cluster “short”)	0.998	0.998	0.998
Conductance Normality *p*‐value (cluster “long”)	0.984	0.984	0.984
Gamma Normality *p*‐value (cluster “short”)	0.999	0.999	0.999
Gamma Normality *p*‐value (cluster “long”)	0.943	0.943	0.943
Conductance t‐statistic	3.772	3.772	3.772
Conductance *t*‐test *p*‐value	0.010	0.010	0.010
Gamma t‐statistic	3.846	3.846	3.846
Gamma *t*‐test *p*‐value	0.009	0.009	0.009
Bootstrap partition frequency	0.778	0.762	0.812
Conductance z‐score	−19.386	−17.276	−16.118
Gamma z‐score	−10.096	−8.484	−9.027

*Note*: Conductance (G) and MO‐electrode coupling (Γ) data from Table 1.

Abbreviation: GMM, Gaussian mixture models.

### 2.4 Machine learning reveals dual transport regimes

Unsupervised ML identified two distinct groups: “short” junctions (n=8–12) and “long” junctions (n=13–15). The “short” group exhibits higher contact conductance ($G_{c,\text{short}}\approx 200\;\mu$S) and steeper attenuation $\left(\beta_{\text{short}}\approx 1.0\right)$, while the “long” group shows lower contact conductance ($G_{c,\text{long}}\approx 8\;\mu$S) and shallower decay $\left(\beta_{\text{long}}\approx 0.74\right)$.

The statistical significance of this bipartition was validated through silhouette scores (0.388), bootstrap resampling (p<0.01 for cluster stability), and consistent grouping across hierarchical, K‐means, and GMM algorithms. Results for G and Γ are visualized in Figure 2 and Supporting Information S1: Figure S6.

### 2.5 Fitting XPS data for SAM thickness uncovers the structural origin of bifurcation

Unsupervised ML identified two distinct groups—“short” (C8A–C12A) and “long” (C13A–C15A) junctions—with differing transport properties (Section 2.3), while quantum chemical calculations revealed nearly isoenergetic planar ($C_{s}$) and skewed ($C_{1}$) conformers (Section 2.1). Assigning these conformers to the ML‐identified groups may be tempting but this claim still poses a challenge: which junctions correspond to which conformers?

XPS data proved as critical as transport measurements in resolving this question, unlike its typical secondary role in molecular junction studies for deriving tilt angles $\theta =\arccos\left(d_{\exp}/L_{\text{estimate}}\right)$ from SAM thickness $d_{\exp}$ computed using measured XPS intensities (Supporting Information S1: Equation (S3), Figures S4 and S5 and Figure 4l,m) and estimated molecular lengths $L_{\text{estimate}}$ (e.g., via ChemBio3D).

**FIGURE 5:**
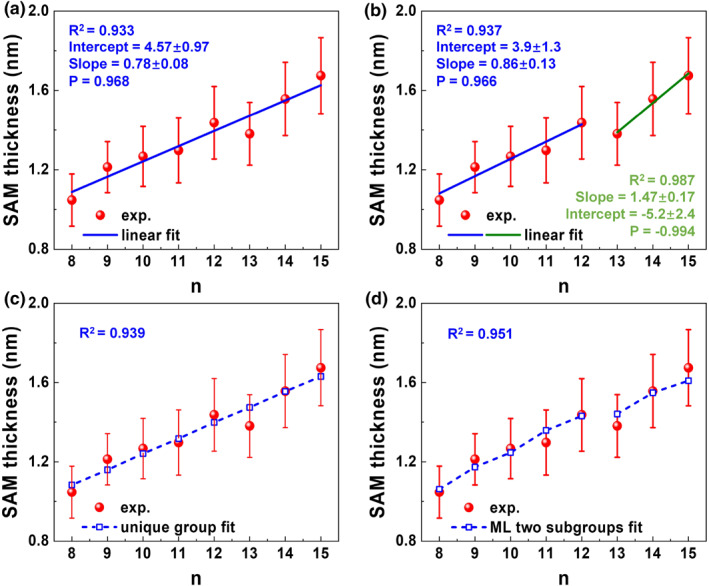
SAM Thickness Analysis Confirms ML‐Identified Conformational Bifurcation in CnA Junctions. Thickness d of CnA SAMs adsorbed on gold computed from XPS‐measured intensities (Supporting Information S1: Equation (S3)) with literature data for attenuation length λ (Supporting Information S1: Equation (S3))^[33]^ as a function of methylene repeat units n was fitted using four models. (a, b), Linear fits assuming either a single homogeneous group (a) or an ML‐guided two‐group model (b). (c, d), Fits based on a detailed structural description of the CnA geometry, incorporating bond lengths and angles from quantum chemical calculations, assuming a single group (c) or two ML‐defined groups (d). The two‐group models (b, d) consistently outperform their single‐group counterparts (a, c), validating the ML‐identified structural dualism (see insets), with further improvements achieved by accounting for atomistic details (compare $R^{2}$ in (b) and (d)). SAM, self‐assembled monolayer.

Initial linear fitting (d=a+bn) showed that ML‐guided bipartition into “short” and “long” groups (Figure 5b) outperforms a single‐group model (Figure 5a). This supports the ML‐identified bifurcation. Although the fact that the slopes substantially differ (0.86 vs. 1.47) is compatible with conformational differences, it does not allow assigning specific conformers to each group.

To address this, we developed a dual‐group fitting strategy for SAM thickness, allowing for distinct conformational regimes while respecting chemical constraints of adsorption on metal. Unlike simplistic linear models, the present theoretical function $d_{n}=d_{n}\left(\theta ,\alpha ,\phi \right)$ uses DFT‐derived bond metrics (M062X/cc‐pVTZ, Gaussian 16^[17]^), detailed in Section S6. It accounts for three parameters: polar angle θ (tilt of the Au–C≡C axis relative to the surface normal), dihedral angle $\alpha =∠\left(C_{2}\text{–}C_{3}\text{–}C_{4}\text{–}C_{5}\right)$ (torsional conformation), and azimuthal angle ϕ (rotation about the C≡C bond while keeping the dihedral angle frozen).

Our theoretical formula $d_{n}=d_{n}\left(\theta ,\alpha ,\phi \right)$ accounts for the fact that the thickness of a given SAM (given n) depends on three parameters: the polar angle θ (tilt of the Au–C≡C axis relative to the surface normal), the dihedral angle $\alpha =∠\left(C_{2}\text{–}C_{3}\text{–}C_{4}\text{–}C_{5}\right)$ (torsional conformation of the alkyl chain), and the azimuthal angle ϕ (rotation of the alkyl backbone about the C≡C bond while keeping the dihedral angle frozen).

The dual‐group fitting minimizes the weighted sum of squared residuals between calculated $\left(d_{\text{calc}}\right)$ and experimental $\left(d_{\exp}\right)$ thicknesses, with goodness‐of‐fit quantified by standard $R^{2}$ metrics (Figure 5d, Table 3). It uses a unique formula for the SAM thickness $d_{n}=d_{n}\left(\theta ,\alpha ,\phi \right)$ with five parameters adapted to the dual‐group strategy: $d_{\text{s,short}}=d_{s}\left(\theta_{\text{shared}},\alpha_{\text{short}},\phi_{\text{short}}\right)$ (s=8,9,10,11,12) and $d_{\text{l,long}}=d_{l}(\theta_{\text{shared}},\alpha_{\text{long}},\phi_{\text{long}})$ (l=13,14,15). The shared θ reflects the near‐linear $\text{Au}\text{–}\;C_{1}\text{–}\;C_{2}\text{–}\;C_{3}$ geometry due to sp‐hybridization of the Au–C bond, which is largely insensitive to chain length or packing density, as supported by prior studies.[^34^, ^35^, ^36^, ^37^, ^38^] This constraint ensures chemical consistency, while distinct α and ϕ account for the ML‐identified structural differences. The dual‐group fit $\left(R^{2}=0.951\right)$ outperforms single‐group (Figure 5c) and linear models (Figure 5a,b).

**TABLE 3:** Dual‐group fitting of the experimental SAM thickness.

CnA	*n*	α	θ	ϕ	$d_{\text{calc}}$	γ	Overall tilt	$d_{\exp}$
C8A	8	175.8	39.2	50.1	10.63	3.83	40.41	10.5±1.3
C9A	9	175.8	39.2	50.1	11.73	3.83	36.58	12.1±1.3
C10A	10	175.8	39.2	50.1	12.47	3.83	40.90	12.7±1.5
C11A	11	175.8	39.2	50.1	13.58	3.83	37.54	13.0±1.6
C12A	12	175.8	39.2	50.1	14.31	3.83	41.28	14.4±1.8
C13A	13	65.0	39.2	14.5	14.41	56.55	34.84	13.8±1.6
C14A	14	65.0	39.2	14.5	15.49	56.55	32.52	15.6±1.9
C15A	15	65.0	39.2	14.5	16.09	56.55	36.54	16.7±1.9

*Note*: Results for the SAM thickness (d) and the relevant angles obtained from dual‐group fitting: θ (polar), ϕ (azimuth), α (dihedral), and γ (angle formed by the triple bond with plane of the carbon backbone of the alkyl chain). Noteworthily, the values of relevant angles characterizing the identical triple bond orientation (polar angle θ, which is common for the two groups) and internal molecular conformation (dihedral angle at the alkyne terminus α=∠(C2−C3−C4−C5), which is different from the two groups, matching the values of the $C_{s}$ and $C_{1}$ conformers in the gas phase). Within experimental errors $\left(d_{\exp}\right)$, the theoretical SAM thickness $\left(d_{\mathrm{calc}}\right)$ are agree well with the experimental values $\left(d_{\exp}\right)$ within errors $\left(\delta d_{\exp}\right)$. Coefficient of determination $R^{2}:0.9514$, lengths in Å, angles in degree.

Most importantly, the resulting dihedral angles—α≈176° for “short” and α≈65° for “long” junctions (Table 3)—match gas‐phase $C_{s}$ (α=180°) and $C_{1}$ (α≈65°) conformers (Section 2.1). This enables unambiguous assignment: “short” junctions adopt planar $C_{s}$ conformers, while “long” junctions adopt skewed $C_{1}$ conformers.

Conformational bifurcation thus drives the dual transport regimes, with alkyne topology influencing contact conductance $\left(G_{c}\right)$ via MO‐electrode coupling $\left(\Gamma_{c}\right)$. The dihedral angle collapse (α≈180°→65°) correlates with a significant conductance reduction $\left(G_{c,\text{short}}/G_{c,\text{long}}\approx 200\;\mu S/8\;\mu S\approx 25\right)$, far exceeding the fivefold change expected from the hypothetical analogy with biphenyl‐based junctions (Equation (2), Section 2.1).

### 2.6 Mechanistic implications

The ML‐identified conformational bifurcation (Section 2.3, Section 2.5) profoundly impacts transport properties in CnA junctions through three key mechanisms:

(i) Internal contact geometry. The planar $C_{s}$ conformers (n=8–12, α≈176°) exhibit stronger electronic coupling between the gold electrode and the acetylenic π‐system (where the dominant transport orbital, HOMO‐1, is localized), resulting in higher contact conductance ($G_{c,\text{short}}\approx 200\;\mu$S) compared to the skewed $C_{1}$ conformers (n=13–15, α≈65°, $G_{c,\text{long}}\approx 8\;\mu$S) (Figure 2b,d). This “internal contact geometry” (the dihedral angle α at the anchor) is distinct from the external molecular orientation on the surface (tilt angle θ).

(ii) Tunneling efficiency. Counterintuitively, the skewed $C_{1}$ conformers reduce the attenuation factor $\left(\beta_{\text{long}}\approx 0.74\right)$ compared to planar conformers $\left(\beta_{\text{short}}\approx 1.02\right)$, enhancing tunneling efficiency over longer chain lengths. Notably, β and the MO energy offset $\varepsilon_{0}$ are independent parameters. Despite the distinct β values, $\varepsilon_{0}$ remains constant within errors across both groups (Supporting Information S1: Figure S3, Table 1), challenging the conventional tunneling barrier model where β is tied to $\varepsilon_{0}$.

(iii) Size‐dependent steric effects. Steric crowding in shorter chains (n≤12) favors planar geometries due to tighter packing in SAMs, while longer chains (n≥13) adopt skewed conformations, likely driven by relaxed steric constraints and optimized dispersion interactions (Section 2.5).

The crossover at n=12–13 reflects a balance between steric constraints and electronic coupling, driving the distinct $G_{c}$ and β values observed in the “short” and “long” groups (Figure 2 and Supporting Information S1: Figure S6). This mechanistic insight, derived from the ML clustering, links molecular conformation to transport properties, highlighting the critical role of alkyne topology in junction performance.

### 2.7 Future perspectives

The conformational duality and dual transport regimes identified in this study open several avenues for further investigation. Complementary techniques could provide additional, direct validation of the proposed bifurcation.

Probing molecular structure and dynamics *in operando*: To obtain direct spectroscopic signatures of the two conformers within an operational junction, **Inelastic Tunneling Spectroscopy (IETS)** would be highly insightful. This technique can act as a “vibrational fingerprint” for molecular conformations. The planar ($C_{s}$) and skewed ($C_{1}$) conformers should exhibit distinct low‐energy torsional and vibrational modes, particularly those involving the alkyne terminus and the adjacent methylene groups. The emergence of two distinct sets of vibrational peaks in IETS spectra, especially for chain lengths near the crossover region (n=12–13), would provide direct, in situ evidence for the coexistence of both conformers under bias.

Directly imaging conformation and packing: A powerful method to independently validate the structural parameters obtained from our dual‐group XPS fitting model is **Sum‐Frequency Generation (SFG) Spectroscopy**. As a surface‐specific technique sensitive to symmetry and orientation, SFG could directly probe the average tilt angle (θ) and, more importantly, the azimuthal angle (ϕ) distribution of the alkyne headgroups on the gold substrate. Experimental confirmation of the significantly different ϕ values for the “short” and “long” (ϕ≈50° and ϕ≈15°, cf. Table 3) groups would provide robust, independent support for the proposed conformational bifurcation and its structural origin.

## 3 CONCLUSION

This study demonstrates that conformational bifurcation drives dual transport regimes in *n*‐alk‐1‐yne molecular junctions. Unsupervised ML robustly partitioned junctions into “short” (C8A–C12A) and “long” (C13A–C15A) groups with distinct transport properties: the “short” group exhibits higher contact conductance $\left(G_{c}\approx 200\;\mu S\right)$ and steeper attenuation (β≈1.0), while the “long” group shows lower conductance $\left(G_{c}\approx 8\;\mu S\right)$ and shallower decay (β≈0.74).

XPS measurements confirmed this bifurcation, revealing dihedral angles of α≈176° for “short” junctions and α≈65° for “long” junctions, matching gas‐phase planar and skewed conformers, respectively.

The independence of the attenuation factor (β) from the MO energy offset $\left(\varepsilon_{0}\right)$, with $\varepsilon_{0}$ remaining nearly constant across both groups (Supporting Information S1: Figure S3), challenges conventional tunneling barrier models according to which $\beta \propto \sqrt{\left|\varepsilon_{0}\right|}$ (providing thereby further support to a similar conclusion based on completely different experimental data^[39]^) and underscores the role of alkyne topology in modulating MO‐electrode coupling $\left(\Gamma_{c}\right)$. Limited to the “short” group, the presently reported bifurcation inherently escaped prior studies on CnA junctions,[^9^, ^40^, ^41^] a fact highlighting the necessity of investigating broader molecular series and advanced analytical methods.

The synergy of unsupervised ML, which efficiently navigated 127 possible bipartitions, with experimental and computational approaches establishes a powerful framework for uncovering hidden patterns in complex nanoscale systems, underscoring the value of data‐driven discovery in materials science.^[42]^ These findings position conformational bifurcation as a fundamental design principle for molecular electronics, enabling tailored transport properties through control of anchoring group orientation. The present ML‐enabled discovery paves the way for advanced nanotechnology applications, including sensors and switches. This methodology provides a blueprint for future investigations of structurally diverse molecular systems, with potential applications in sensors, switches, and other molecular‐scale devices.

## 4 METHODS

### 4.1 Experimental methods

Materials: Gold nuggets (99.999% pure) evaporation boats and chromium evaporation rod were purchased from Kurt J. Lesker Co. Contact mode Atomic Force Microscope (AFM) tips (DNP‐10 silicon nitride probes) were purchased from Bruker AFM Probes. 1‐decyne (C8A, 98%), 1‐undecyne (C9A, 98%), 1‐dodecyne (C10A, 98%), 1‐tetradecyne (C12A, 97%) and 1‐Pentadecyne (C13A, 97%) used in this study were purchased from Sigma‐Aldrich Company. 1‐tridecyne (C11A, 95%) was purchased from Aladdin company. 1‐hexadecyne (C14A, 97%) and 1‐heptadecyne (C15A, 97%) were purchased from Tokyo Chemical Industry company.

Conducting tip and sample preparation: Preparing conductive AFM tips. Contact‐mode AFM tips were coated with Au using a thermal evaporator. The evaporation process was conducted inside a nitrogen‐filled glovebox, maintaining low levels of water and oxygen (both <0.1 ppm). A 500 Å metal layer (Au) was deposited onto a 50 Å chromium (Cr) adhesion layer, at a deposition rate of 0.5–1.0 Å/s. After deposition, the coated tips were immediately transferred without exposure to air to another glovebox containing the CP‐AFM setup for conductance measurements.

Preparation of flat metal substrates. Template‐stripped flat metal substrates were employed to grow high‐quality SAMs. For flat Au substrates: A 5000 Å thick layer of Au was deposited onto clean Si wafers using a thermal evaporator. Next, silicon chips (1 ${\text{cm}}^{2}$) were affixed onto the metal surface using epoxy (EPOTEK 377, Epoxy Technologies, MA). The epoxy layer was cured by placing the wafers in an oven at 120 °C for 1.5 h. SAMs were formed by immersing template‐stripped flat metal substrates into 1 mM ethanol and alkyne solutions of the molecules individually for 20 h. After rinsing with sufficient ethanol and drying with nitrogen flow, the samples were ready for measurements.

Angle resolved XPS measurements: The angle resolved XPS measurements were performed on a PHI Versa Probe III system (ULVAC‐PHI) utilizing a monochromated Al Kα X‐ray source (1486.6 eV). The analysis chamber's base pressure was maintained at $5.0\times 10^{-8}$ Pa, with a working pressure of approximately $1.0\times 10^{-6}$ Pa during data acquisition. The X‐ray source was operated with a 200μm spot size, at 25 W power and 15 kV. Core‐level spectra for C 1s, S 2p, and Au 4f were collected using a pass energy of 112 eV, a step size of 0.1 eV, and a dwell time of 20 ms per step. Angle‐resolved measurements were performed by setting the angle between the sample surface and the analyzer axis to 20°, 40°, 60°, and 80°.

Transport measurements: The prepared flat metal substrates with SAMs were securely mounted in the AFM. Conductive AFM tips, coated with Au as previously described, were brought into contact with the SAM surface by applying a compressive load of approximately 1 nN.

The resulting junction structure, with molecules forming a stable physisorptive contact with the tip at the methyl terminus[^13^, ^43^, ^44^] and chemisorbed on the bottom electrode via the acetylenic group,[^9^, ^40^, ^41^] is a well‐established configuration in molecular electronics. The exponential decay of conductance with molecular length (Figure 2) confirms a non‐resonant tunneling mechanism through the molecular backbone, providing a definitive signature of robust molecular junction formation.^[9]^

A DC voltage was applied to the tip using a Keithley 2635B source‐measure unit operated in DC mode. Current–voltage (I–V) curves were recorded as the voltage was swept across the junction. Applied voltage was swept between ±1.5 V.

### 4.2 Theoretical methods

Quantum chemical calculations: Geometries for the nearly isoenergetic planar and skewed conformers of isolated CnA molecules were optimized using Gaussian 16 at the M062X/cc‐pVTZ level of theory with Grimme's GD3 dispersion correction.^[17]^ The planar $\left(C_{s}\right)$ and skewed $\left(C_{1}\right)$ conformers were confirmed as minima through vibrational frequency analysis.

Transport modeling: The low‐bias junction conductance G was determined from the slope (dI/dV) of the linear region (±0.1 V) of the I–V curves.

The effective MO‐electrode coupling Γ was calculated with the MO energy offset $\varepsilon_{0}$ derived from transition voltage spectroscopy (Supporting Information S1: Equation (S2)) based on the off‐resonant single level model.^[24]^

Machine learning analysis: To objectively assess potential grouping in molecular transport data, we employed three unsupervised ML methods.

Clustering methods include: Hierarchical clustering (Ward's linkage)^[28]^ to minimize within‐cluster variance, K‐means clustering^[30]^ with k=2 centroids (10 random initializations), and GMM^[32]^ with expectation‐maximization for probabilistic assignments.

Validation framework relied on: silhouette analysis for cluster cohesion/separation, bootstrap resampling (100 iterations) for robustness, and synthetic dataset validation (see Supporting Information S1: Section S4).

All analyses were implemented in Python using scikit‐learn,[^45^, ^46^] with details of numerical results and statistical significance presented in Section 2.

## CONFLICT OF INTEREST STATEMENT

The authors declare no conflicts of interest.

## ETHICS STATEMENT

No animal or human experiments were involved in this study.

## Supporting information

Supporting Information S1

## Data Availability

Data available on request from the authors.
