# Supplementary material for: Conformational bifurcation drives dual transport regimes in molecular junctions: Unsupervised machine learning insights
Source: Smart Mol. 2026 Jan 17;4(1):e70033. doi: 10.1002/smo2.70033 (PMC13104088; doi:10.1002/smo2.70033)
Supplement: Supplementary file 1 — Supporting Information S1 [file SMO2-4-e70033-s001.pdf]

Supporting Information — Conformational Bifurcation Drives Dual Transport Regimes in Molecular Junctions: Unsupervised Machine Learning Insights

Yuhong Chen, Ioan Bâldea, Elad Koren, Zuoti Xie**

Yuhong Chen
Department of Materials Science and Engineering, MATEC, Guangdong Technion-Israel Institute of Technology, 241 Daxue Road, Shantou, Guangdong, 515063, China
Dr. Ioan Bâldea
Theoretical Chemistry, Heidelberg University, Im Neuenheimer Feld 229, D-69120 Heidelberg, Germany
Email Address: ioan.baldea@pci.uni-heidelberg.de
Prof. Elad Koren
Department of Materials Science and Engineering, Technion-Israel Institute of Technology, Haifa 3200003, Israel
Prof. Zuoti Xie
Department of Materials Science and Engineering, Technion-Israel Institute of Technology, Haifa 3200003, Israel
Department of Materials Science and Engineering, MATEC, Guangdong Technion-Israel Institute of Technology, 241 Daxue Road, Shantou, Guangdong, 515063, China
Quantum Science Center of Guangdong-Hong Kong-Macao Greater Bay Area (Guangdong), Shenzhen-Hong Kong International Science and Technology Park, NO.3 Binglang Road, Futian District, Shenzhen, Guangdong, 518000, China
Email address: zuoti.xie@gtiit.edu.cn

Keywords: *Molecular Electronics, Charge Transport, Molecular Junctions, Conformational Bifurcation, Structure-Property Relationships, n-Alk-1-Ynes*

S1 Isolated CnA Molecules

Geometries provided by ChemBio 3D and resulted from our DFT calculations using M062x/cc-pVTZ with GD3 empirical dispersion corrections as implemented in GAUSSIAN 16 [1] (ref. [1] displays the complete list of authors of ref. 14 in the main text) are depicted in Figure S1. The latter refer to the planar C_s conformer of the "short" species ($8 \le n \le 12$) and to the skewed C_1 conformer of the "long" species ($13 \le n \le 15$) reflecting the dual transport regime and the conformational bifurcation found in experiment.

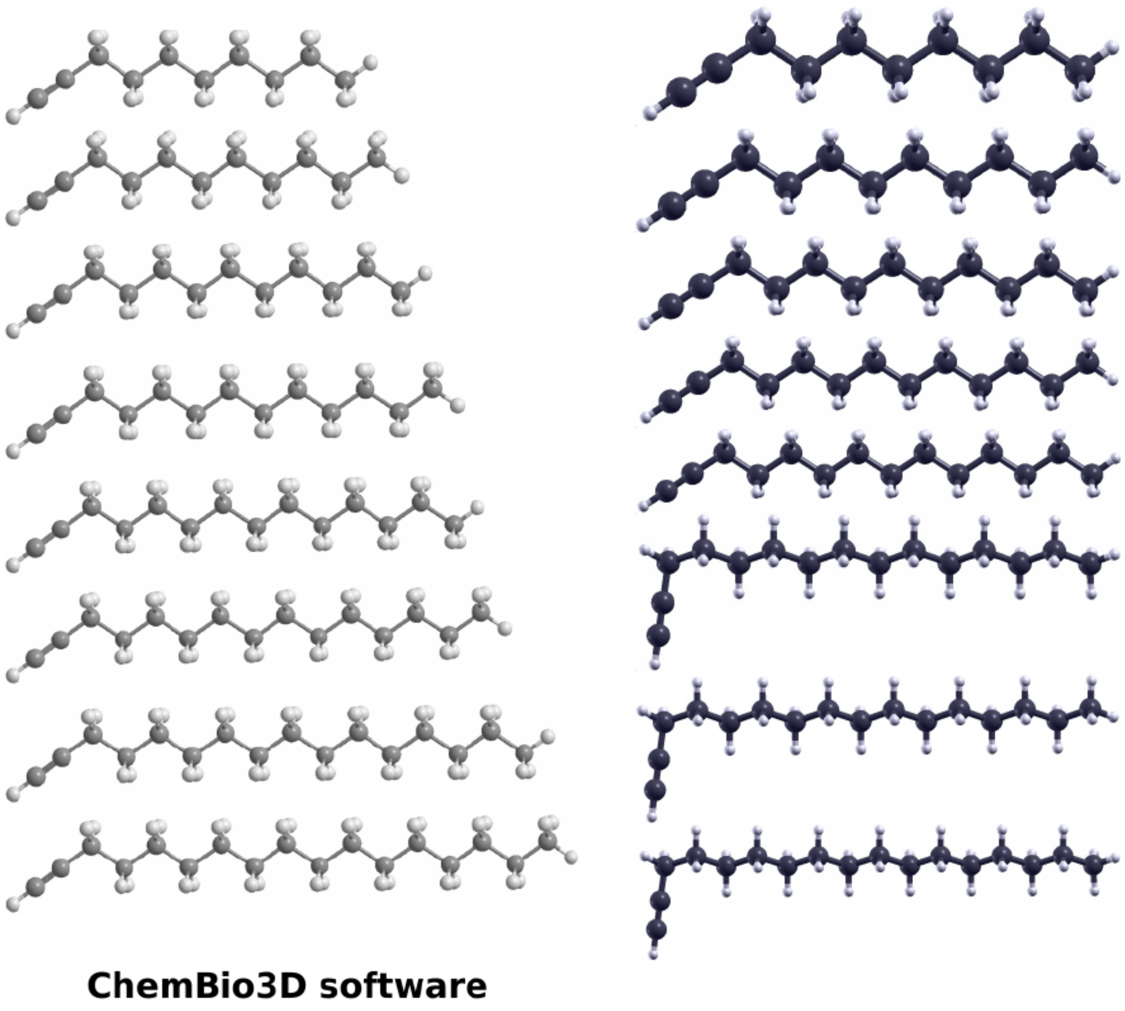

Figure S1: **Conformational Geometries of Isolated CnA Molecules.** Left: Planar C_s conformers for C8A to C15A (1-decyne to 1-heptadecyne) generated by ChemBio3D. Right: DFT-optimized (M06-2X/cc-pVTZ with GD3 corrections [1]) conformers depicted using XCrySDen [2]. The optimized structures match the dual-group assignment from the SAM thickness analysis (Section 3.5): C_s planar conformers for the "short" group (C8A–C12A) and C_1 skewed conformers for the "long" group (C13A–C15A).

S2 Off-Resonant Single Level Model of Transport

At low temperatures and far from resonance, conditions satisfied by CnA junctions [3], the current (I) and low-bias conductance (G) through a junction with N molecules mediated by a single molecular orbital

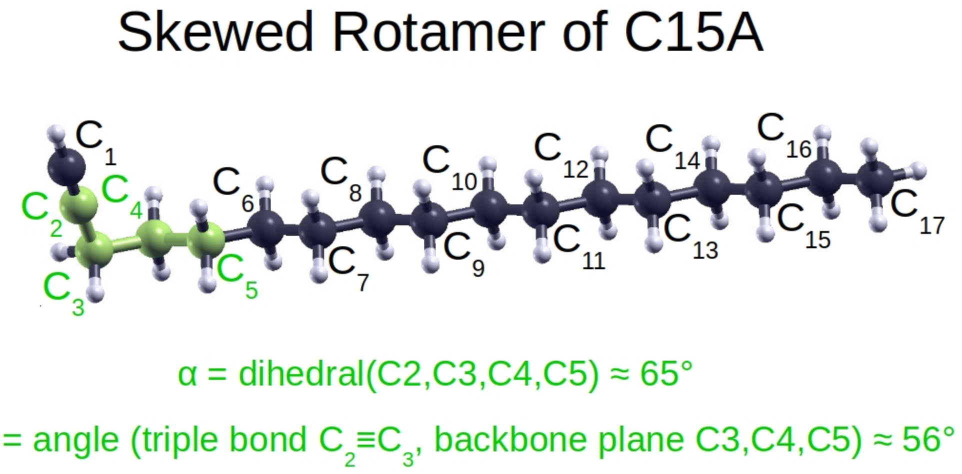

Figure S2: **Illustrating the Salient Structural Features of the Skewed C_1 Rotamer.** The structure of the skewed C_1 conformer of C15A, with carbon atoms numbered using IUPAC convention (C1 at the alkyne terminus). The figure highlights the dihedral angle at the alkyne terminus, $\alpha = \angle(C_2{-}C_3{-}C_4{-}C_5) \approx 65°$, and the angle of $\approx 56°$ between the triple bond $C_2{\equiv}C_3$ and the plane of the alkyl's carbon backbone defined by the atoms (C_3, C_4, C_5). These angles, derived from quantum chemical calculations (M062X/cc-pVTZ with GD3 dispersion corrections [1]) and shown in the inset, characterize the conformational bifurcation driving the distinct transport properties of "long" junctions (Section 3.5, Table 3). Figure generated using XCrySDen [2].

where $G_0 = 2e^2/h = 77.48\,\mu$S is the quantum conductance, $\Gamma = \sqrt{\Gamma_s \Gamma_t}$ is the effective MO-electrode coupling (mathematically defined as the geometric average of the electronic couplings of the dominant molecular orbital (HOMO-1 in the case of CnA [3]) to the substrate (Γ_s) and tip (Γ_t) electrodes), and $N = 80$ is the number of molecules per junction, consistent with prior work [3]. The MO energy offset ε_0 is nearly chain-length-independent (Figure S3), and this relates the decay coefficient $\overline{\beta} \approx \beta/2$ to conductance decay (eq (1)).Eq (**??**) accurately reproduces measured I–V curves (Figures 2d–l).

Noteworthily, the number of molecules per junction (for which we used the value $N = 80$ consistent to earlier work [3]) merely acts as a multiplicative factor in eq (**??**). This dependence on N confers robustness to the present conclusions not only for G (whose values are model independent) but also for Γ. Possible inaccuracies in N merely rigid vertical shifts of the plots for Γ (Figures 3c and d) without any impact on the values of the exponential attenuation factors ($\overline{\beta}$).

An important advantage of the above model is the fact that it allows to easily estimate the the MO energy offset $|\varepsilon_0|$ can be determined from the peak location of the quantity $V^2/|I|$ at positive and negative biases, which define the transition voltages V_{t+} and V_{t-} (Figure 2c).

$$|\varepsilon_0| = \frac{2eV_{t+}\,|V_{t-}|}{\sqrt{V_{t+}^2 + 10V_{t+}\,|V_{t-}| + V_{t-}^2}} \tag{S2}$$

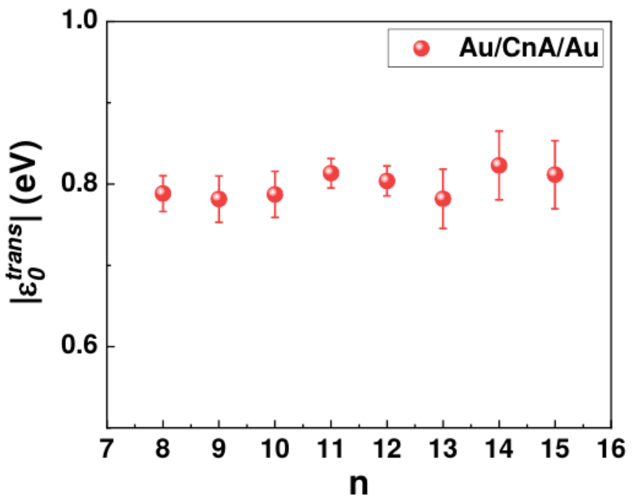

Figure S3: **MO Energy Offset from Transition Voltage Spectroscopy.** ε_0 values for CnA junctions (n = 8–15) derived from eq (S2).

The values of the MO energy offset obtained from eq (S2) are collected in Table 1 and depicted in Figure S3. They were used to compute the values of the effective MO-electrode coupling Γ via eq (**??**) included in Table 1.

S3 Complementary Details on Machine Learning

To make the information that follows accessible to molecular electronics researchers unfamiliar with ML, similar to the main text, we draw below analogies to general chemical concepts.

S3.1 Method Selection Rationale

We selected the methods described in the main text (hierarchical and k-means clustering, and Gaussian Mixture Models (GMM)), because:

- No Pre-labeled Data Required: Unsupervised ML identifies patterns without prior group knowledge, [5] similar to analyzing unknown molecules.
- Suitable for Small Datasets: These methods perform well with eight data points, unlike complex ML requiring thousands of points [6].
- Interpretable Results: Outputs (e.g., tree diagrams, group centers) are intuitive, [7] like visualizing molecular clusters.
- Diverse Approaches: Hierarchical and K-means group by measurement similarity (like molecular distances), while GMM uses probabilistic assignments (like reaction likelihoods) [8].

Other methods, such as DBSCAN [9] or neural networks [10], require larger datasets or focus on visualization (e.g., t-SNE [11]), making them less suitable for this grouping task.

S3.2 Methods Suitability

Hierarchical and K-means are well-suited for small datasets, akin to sorting a few molecules by conductance. GMM is better for larger datasets but still applicable with adjustments. Unsupervised ML is ideal

for exploring patterns without predefined groups. We evaluated reliability using silhouette scores (measuring group separation, like purity in chromatography), agreement scores (comparing method consistency), statistical tests (normality and mean comparisons), and bootstrap resampling (like repeating experiments).

S3.3 Group Quality

A silhouette score of 0.388 (scale 0 to 1) indicates robust group separation for a small dataset of eight points, comparable to a clean chromatographic separation [12]. This score, combined with an Adjusted Rand Index (ARI) of 1.000 confirming identical groupings across methods [13], supports two distinct transport regimes, as visualized in Figures 1b,d. Table 2 summarizes these findings.

S3.4 Trends and Statistical Tests

Logarithmic fits analyzed trends in $\ln(G)$ and $\ln(\Gamma)$, akin to tracking reaction rates. Group 0 exhibited steeper decays ($\ln(G)$: -1.020, $\ln(\Gamma)$: -0.499) than Group 1 ($\ln(G)$: -0.738, $\ln(\Gamma)$: -0.348), with $R^2 \geq 0.993$, indicating excellent fits (see Figures 1b,d). Z-scores ($\ln(G)$: -7.551, $\ln(\Gamma)$: -5.869, $p < 0.001$) confirmed significant slope differences. Shapiro-Wilk tests verified normality ($\ln(G)$: Cluster “short” W=0.997, p=0.998; Cluster “long” W=1.000, p=0.984; $\ln(\Gamma)$: Cluster “short” W=0.999, p=0.999; Cluster “long” W=0.999, p=0.943), enabling Welch’s t-tests, which showed significant mean differences ($\ln(G)$: $t = 3.772$, $p = 0.010$; $\ln(\Gamma)$: $t = 3.846$, $p = 0.009$). These results confirm distinct transport properties in the “short” and “long” groups.

S3.5 Bootstrap Stability

Bootstrap resampling (100 iterations with data variations, like repeated experiments) demonstrated robust clustering, with group frequencies of 77.8% (Hierarchical), 76.2% (K-means), and 81.2% (GMM). Consistent z-scores (e.g., Hierarchical $\ln(G)$ -19.386, $\ln(\Gamma)$ -10.096) further validate the reliability of the identified transport regimes.

S4 Machine Learning Analysis of the Synthetic Dataset

The synthetic data, generated with exact parameters derived from Table 1, closely mimic the real data’s structure, with a slightly higher silhouette score and larger z-score magnitudes due to reduced noise. Hierarchical clustering uses a dendrogram-based approach, K-means minimizes within-cluster variance, and GMM employs probabilistic modeling. These methods were chosen for their interpretability, robustness to small datasets, and unsupervised nature. Both datasets show robust clustering (silhouette scores 0.388–0.430, significant slope differences), validating two distinct subgroups.

The synthetic data, generated with exact parameters from Table 1, closely replicate the real data’s cluster structure, with minor deviations due to controlled noise levels. They confirm the reliability of our methods (details in Section 3.3).

S4.1 Analysis

The robust partition and significant z-scores validate two distinct subgroups, likely reflecting physical differences. Bootstrap frequencies confirm stability, with consistent z-scores supporting subgroup detection.

S4.2 Results for Synthetic Data

The synthetic dataset (8 points) was generated with exact slopes and intercepts derived from Table 1, closely mimicking the real data's structure. A slightly higher silhouette score and larger z-score magnitudes result from reduced noise levels (standard deviations of 0.02 for G and 0.005 for Γ).

S4.3 Clustering Quality

Silhouette scores of 0.430 indicate strong separation, exceeding real data (0.388). ARI = 1.000 confirms consistent partitions. Clusters were $\{0 : [9, 12, 11, 10, 8], 1 : [13, 14, 15]\}$, matching real data's structure. Table S1 summarizes metrics.

S4.4 Regression and Statistical Comparison

Slopes closely align with real data: Cluster "short" ($G : -1.019$, Γ: -0.499, $R^2 > 0.999$), Cluster "long" ($G : -0.714$, $\Gamma : -0.347$, $R^2 > 0.999$). Z-scores ($G : -34.536$, $\Gamma : -35.955$, $p < 0.001$) are significant and negative, consistent with real data, though larger due to tighter fits.

S4.5 Bootstrap Stability

Partition frequencies (0.500–0.759) approach real data (0.762–0.812), indicating robust clustering. Z-scores are negative but inflated (e.g., Hierarchical $G : -110.669$), reflecting high R^2 values and small sample variability.

Table S1: **Clustering and Regression Results for Synthetic Data.** Synthetic data for conductance (G) and MO-Electrode coupling (Γ) based on real data from Table 1.

Metric	Hierarchical	K-means	GMM
Silhouette Score	0.430	0.430	0.430
ARI (vs. Hierarchical)	—	1.000	1.000
Cluster "short" Size (n values)	5 ([8, 9, 10, 11, 12])	Same	Same
Cluster "long" Size (n values)	3 ([13, 14, 15])	Same	Same
Cluster "short" G Slope (R^2)	-1.019 ecs(0.999)	Same	Same
Cluster "long" G Slope (R^2)	-0.714 (0.999)	Same	Same
Cluster "short" Γ Slope (R^2)	-0.499 (0.999)	Same	Same
Cluster "long" Γ Slope (R^2)	-0.347 (0.999)	Same	Same
G Z-score (p-value)	-34.536 (< 0.001)	Same	Same
Γ Z-score (p-value)	-35.955 (< 0.001)	Same	Same
Bootstrap Partition Frequency	0.704	0.759	0.500
Bootstrap G Z-score	-110.669	152.695	-84.693
Bootstrap Γ Z-score	-44.054	-36.468	-29.024

S5 Additional Angle Resolved XPS Data

To estimate the SAM thickness d of the CnA SAMs, we utilized angle-resolved XPS. The value of d was calculated using the formula

$$d = \lambda \sin \delta \ln \left(\frac{I}{I_0} \right) \quad \text{(S3)}$$

where I_0 is the photoelectron intensity from the bare gold substrate, I is the intensity from the SAM-coated substrate, $\lambda = 4.2\,\text{nm}$ is the attenuation length [14], and δ $(= 20°, 40°, 60°, 80°)$ is the takeoff angle between

the gold surface and analyzer axis. The angles in parentheses represent those at which signals for Au 4f and C 1s core-level photoelectrons were collected (Figures S4 and S5).

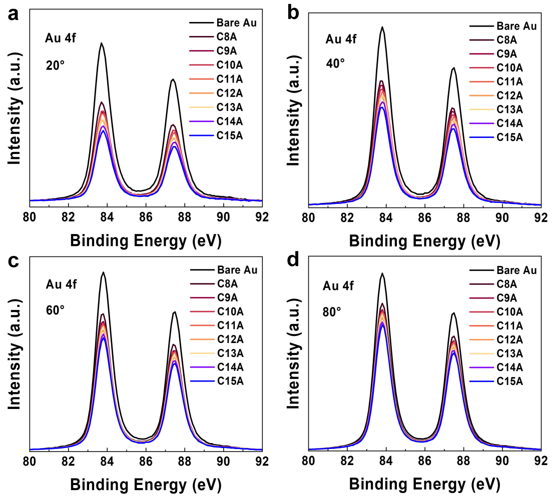

Figure S4: **Au 4f XPS Spectra for CnA on Gold.** Data collected at takeoff angles shown in the inset: (a) $\delta = 20°$, (b) $\delta = 40°$, (c) $\delta = 60°$, (d) $\delta = 80°$.

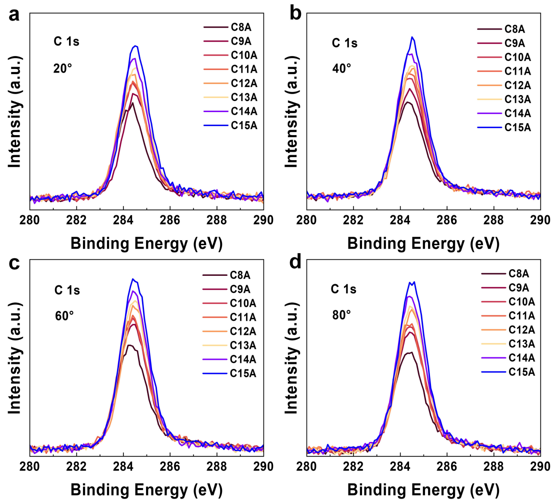

Figure S5: **C 1s XPS Spectra for CnA on Gold.** Data collected at takeoff angles shown in the inset: (a) $\delta = 20°$, (b) $\delta = 40°$, (c) $\delta = 60°$, (d) $\delta = 80°$.

S6 Details on Fitting the Experimental Data for SAM Thickness

To fit the X-ray photoelectron spectroscopy (XPS) data for self-assembled monolayer (SAM) thickness, we developed a theoretical model for n-alk-1-yne (CnA) molecules, avoiding a simplistic linear dependence ($d_n = a + bn$). The model represents each CnA molecule as two moieties (IUPAC numbering, starting from the triple bond, cf. Figure S2): a linear alkyne terminus ($Au{-}C_1{\equiv}C_2{-}C_3$) and an all-trans alkyl backbone ($C_3{-}C_4{-}\ldots{-}C_{n+2}H_3$). The terminal methyl group ($-CH_3$) is modeled with tetrahedral geometry, where the furthest hydrogen atom defines the SAM thickness.

The geometric parameters, averaged from DFT calculations (M06-2X/cc-pVTZ with GD3 dispersion corrections [15] using Gaussian 16 suite [1]), are nearly chain-length-independent (n = 8–15):

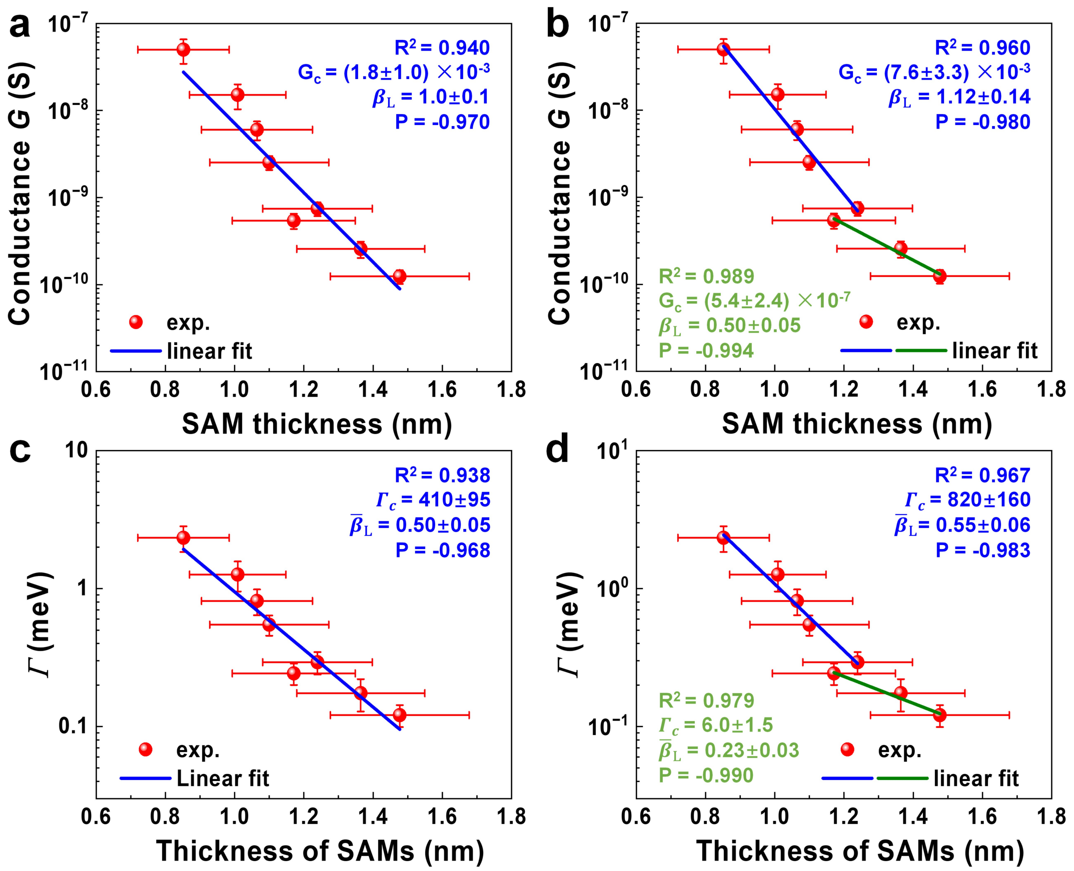

Figure S6: **Transport Properties versus SAM Thickness.** (**a,b**) Low-bias conductance (G) and (**c,d**) MO-electrode coupling (Γ) versus SAM thickness (Z), confirming the ML-identified bifurcation into "short" (n = 8–12) and "long" (n = 13–15) groups (Figure 3). Insets show extracted parameters (notation as in Figure 3), reinforcing dual transport regimes.

- Triple bond: $d(\mathrm{C_1{\equiv}C_2}) = d_{\text{C-C triple}} \approx 1.20\,\text{Å}$.
- Single bond near triple bond: $d(\mathrm{C_2{-}C_3}) = d_{\text{C-C near triple}} \approx 1.44\,\text{Å}$.
- Alkyl chain single bond: $d(\mathrm{C}_i{-}\mathrm{C}_{i+1}) = d_{\text{C-C alkane}} \approx 1.54\,\text{Å}$.
- Alkyl chain angle: $\angle(\mathrm{C}_{k-1}{-}\mathrm{C}_k{-}\mathrm{C}_{k+1}) \approx 113°$.
- C–H bond: $d(\mathrm{C}_{n+2}{-}\mathrm{H}) = d_{C-H} \approx 1.09\,\text{Å}$.
- Methyl group angles: $\angle(\mathrm{H}{-}\mathrm{C}_{n+2}{-}\mathrm{H}) \approx 108°$, $\angle(\mathrm{C}_{n+1}{-}\mathrm{C}_{n+2}{-}\mathrm{H}) \approx 111°$.
- Substrate interface: $d(\mathrm{Au{-}C_1}) = d_{\text{Au-C}} \approx 2.0\,\text{Å}$.

These parameters were treated as fixed, an excellent approximation given the experimental uncertainties in SAM thickness measurements (Table 1). The dihedral angle $\alpha = \angle(\mathrm{C_2{-}C_3{-}C_4{-}C_5})$, along with the polar angle θ (tilt of the Au−C≡C axis relative to the surface normal) and azimuthal angle φ (rotation of the alkyl backbone about the C≡C bond while keeping the dihedral angle frozen), were determined via dual-group fitting to XPS data, as detailed in Section 3.5. This fitting revealed planar (C_s, $\alpha \approx 176°$) and skewed (C_1, $\alpha \approx 65°$) conformers, confirming the structural basis for the observed transport bifurcation.

Formula for the SAM Thickness $\boldsymbol{d} = f(\boldsymbol{\theta}, \boldsymbol{\alpha}, \boldsymbol{\phi})$Formula for the SAM Thickness

The theoretical SAM thickness d is defined as the maximum z-coordinate among the hydrogen atoms (H_1, H_2, H_3) of the terminal CH_3 group in an n-alk-1-yne (CnA) molecular junction, relative to the gold atom at the origin, which belongs to the metal surface at $z = 0$.

Below, we derive $d = z_{\text{H,max}} = f(\theta, \alpha, \phi)$ as a function of the polar angle θ (angle of the $\text{Au}-\text{C}_1-\text{C}_2-\text{C}_3$ unit relative to the surface normal, i.e., the z-axis), the dihedral angle $\alpha = \angle(\text{C}_2-\text{C}_3-\text{C}_4-\text{C}_5)$, and the azimuth angle ϕ (rigid rotation of the alkyl chain around the C1-C2-C3 axis). The derivation uses the bond lengths and angles from the computational model: $d_{\text{C-C alkane}} = 1.54\,\text{Å}$, $d_{\text{C-C near triple}} = 1.44\,\text{Å}$, $d_{\text{C-C,triple}} = 1.2\,\text{Å}$, $d_{\text{Au-C}} = 2.0\,\text{Å}$, $\alpha_{\text{CCC alkane}} = 113°$, $d_{\text{C-H}} = 1.09\,\text{Å}$, $\alpha_{\text{CCH}} = 113°$, and $\alpha_{\text{HCH}} = 105.7°$.

Step 1: Backbone Carbon Coordinates

The alkane chain of the CnA molecule (carbon atoms C_3 to C_{n+2}) is initially constructed starting from the carbon atom C_{n+2} of the end metylene unit (CH_3) in the xy-plane with an all-trans configuration. For carbon $i \in \{3, \ldots, n+2\}$, the coordinates before rotations are:

$$\text{For odd } i = 2k-1, \quad \mathbf{r}_{\text{C}i} = \left((2k-3)\alpha_x, \alpha_y, 0\right),$$
$$\text{For even } i = 2k, \quad \mathbf{r}_{\text{C}i} = \left(2(k-1)\alpha_x, 0, 0\right),$$

where $\alpha_x = d_{\text{C-C alkane}} \sin\left(\frac{\alpha_{\text{CCC alkane}}}{2}\right) = 1.54 \sin(56.5°) \approx 1.2817\,\text{Å}$, and $\alpha_y = d_{\text{C-C alkane}} \cos\left(\frac{\alpha_{\text{CCC alkane}}}{2}\right) = 1.54 \cos(56.5°) \approx 0.8484\,\text{Å}$.

For $n \geq 3$, C_2 is placed relative to C_3, accounting for the distinct C_2-C_3 bond length:

$$\mathbf{r}_{\text{C2}} = \mathbf{r}_{\text{C3}} - \mathbf{R}_{\text{C3-C4}}(\alpha - 180°) \cdot \left(d_{\text{C-C near triple}} \frac{\mathbf{r}_{\text{C3}} - \mathbf{r}_{\text{C2,initial}}}{\|\mathbf{r}_{\text{C3}} - \mathbf{r}_{\text{C2,initial}}\|} \right),$$

where $\mathbf{R}_{\text{axis}}(\psi)$ is the rotation matrix around axis **axis** by angle ψ, and $\mathbf{r}_{\text{C2,initial}} = \mathbf{r}_{\text{C3}} + (d_{\text{C-C near triple}}, 0, 0)$.

The positions of C_1 and Au atoms are

$$\mathbf{r}_{\text{C1}} = \mathbf{r}_{\text{C2}} + d_{\text{C-C,triple}} \frac{\mathbf{r}_{\text{C2}} - \mathbf{r}_{\text{C3}}}{\|\mathbf{r}_{\text{C2}} - \mathbf{r}_{\text{C3}}\|}, \quad \mathbf{r}_{\text{Au}} = \mathbf{r}_{\text{C1}} + d_{\text{Au-C}} \frac{\mathbf{r}_{\text{C2}} - \mathbf{r}_{\text{C3}}}{\|\mathbf{r}_{\text{C2}} - \mathbf{r}_{\text{C3}}\|}.$$

All coordinates are shifted so $\mathbf{r}_{\text{Au}} = (0, 0, 0)$.

Step 2: Polar Rotation

The molecule is rotated to align the C_1-C_2 vector with polar angle θ relative to the z-axis. The C_1-C_2 vector has length $l_{\text{C1-C2}} = d_{\text{C-C,triple}} = 1.2\,\text{Å}$. The desired C_1-C_2 vector is:

$$\mathbf{v}_{\text{C1-C2,desired}} = l_{\text{C1-C2}} \left(\sin\theta\cos\phi, \sin\theta\sin\phi, \cos\theta\right).$$

The rotation matrix $\mathbf{R}_{\text{polar}}$ aligns $\mathbf{r}_{\text{C2}} - \mathbf{r}_{\text{C1}}$ to $\mathbf{v}_{\text{C1-C2,desired}}$. For each atom, the new coordinates are:

$$\mathbf{r}'_i = \mathbf{R}_{\text{polar}} \cdot \mathbf{r}_i.$$

Step 3: Azimuth Rotation

For $n \geq 3$, carbons C_4 to C_{n+2} are rotated around the C_2-C_3 axis by azimuth angle ϕ. The axis is $\mathbf{v}_{\text{C2-C3}} = \mathbf{r}'_{\text{C3}} - \mathbf{r}'_{\text{C2}}$, and the rotation matrix is $\mathbf{R}_{\text{C2-C3}}(\phi)$. Coordinates for $i \geq 4$ become:

$$\mathbf{r}''_{\text{C}i} = \mathbf{R}_{\text{C2-C3}}(\phi) \cdot (\mathbf{r}'_{\text{C}i} - \mathbf{r}'_{\text{C2}}) + \mathbf{r}'_{\text{C2}}, \quad \mathbf{r}''_{\text{C2}} = \mathbf{r}'_{\text{C2}}, \quad \mathbf{r}''_{\text{C1}} = \mathbf{r}'_{\text{C1}}, \quad \mathbf{r}''_{\text{Au}} = \mathbf{r}'_{\text{Au}}.$$

Step 4: Terminal CH_3 Hydrogens

For $n \geq 4$, the terminal CH_3 group is placed at C_{n+2} using $\mathbf{r}''_{C_{n+2}}$, $\mathbf{r}''_{C_{n+1}}$, and $\mathbf{r}''_{C_n}$. The vector $\mathbf{v}_{\text{bond}} = \frac{\mathbf{r}''_{C_{n+2}} - \mathbf{r}''_{C_{n+1}}}{\|\mathbf{r}''_{C_{n+2}} - \mathbf{r}''_{C_{n+1}}\|}$ defines the C-C bond direction. The plane normal is $\mathbf{n}_{\text{plane}} = \frac{\mathbf{v}_{\text{prev}} \times \mathbf{v}_{\text{bond}}}{\|\mathbf{v}_{\text{prev}} \times \mathbf{v}_{\text{bond}}\|}$, where $\mathbf{v}_{\text{prev}} = \frac{\mathbf{r}''_{\text{Cn-1}} - \mathbf{r}''_{\text{Cn-2}}}{\|\mathbf{r}''_{\text{Cn-1}} - \mathbf{r}''_{\text{Cn-2}}\|}$. The in-plane perpendicular vector is $\mathbf{v}_{\text{perp}} = \mathbf{n}_{\text{plane}} \times \mathbf{v}_{\text{bond}}$. The hydrogen H_1 position is

$$\mathbf{r}_{\text{H1}} = \mathbf{r}''_{C_{n+2}} - \left(d_{\text{C-H}} \cos \alpha_{\text{CCH}} \mathbf{v}_{\text{bond}} + d_{\text{C-H}} \sin \alpha_{\text{CCH}} \mathbf{v}_{\text{perp}}\right).$$

The center (M) of the triangle of the three H atoms of the CH_3 group lies along $\mathbf{v}_{\text{bond}}$:

$$\mathbf{r}_{\text{M}} = \mathbf{r}''_{C_{n+2}} - d_{\text{C}-\text{H}} \cos \alpha_{\text{CCH}} \mathbf{v}_{\text{bond}}.$$

Hydrogens H_2 and H_3 are placed at $\pm 60°$ around $\mathbf{v}_{\text{bond}}$

$$\mathbf{r}_{\text{H2,H3}} = \mathbf{r}_{\text{M}} + d_{\text{C-H}} \sin \alpha_{\text{CCH}} \left(\cos(\pm 60°)\mathbf{v}_{\text{perp}} + \sin(\pm 60°)(\mathbf{v}_{\text{bond}} \times \mathbf{v}_{\text{perp}})\right).$$

The SAM thickness is the largest z-coordinate of the three H atoms arond the terminal carbon C_{n+2}

$$d = z_{\text{H,max}} = \max\{z_{\text{H}_1}, z_{\text{H}_2}, z_{\text{H}_3}\}.$$

Due to the complex dependence on θ, α, and ϕ, the exact $d = f(\theta, \alpha, \phi)$ used for fitting the experimental SAM thickness deduced from measured XPS intensities (Figures 2l,m, S4, S5 and eq (S3)) requires numerical computation of hydrogen coordinates.

Table S2: **Single-Group Fitting of the Experimental SAM Thickness.** Results for the SAM thickness (d) and the relevant angles obtained from data fitting assuming CnA junctions forming a homogeneous group: θ (polar), ϕ (azimuth), α (dihedral), and γ (angle formed by the triple bond with plane of the carbon backbone of the alkyl chain). Lengths in Å, angles in degree. Coefficient of determination R^2 : 0.9394.

CnA	n	α	θ	ϕ	d_{calc}	γ	Overall Tilt	d_{exp}
C8A	8	112.322	8.191	37.8	10.844615	58.378	33.219	10.5 ± 1.3
C9A	9	112.322	8.191	37.8	11.597240	58.378	35.092	12.1 ± 1.3
C10A	10	112.322	8.191	37.8	12.414337	58.378	36.528	12.7 ± 1.5
C11A	11	112.322	8.191	37.8	13.166962	58.378	37.852	13.0 ± 1.6
C12A	12	112.322	8.191	37.8	13.984059	58.378	38.879	14.4 ± 1.8
C13A	13	112.322	8.191	37.8	14.736684	58.378	39.864	13.8 ± 1.6
C14A	14	112.322	8.191	37.8	15.553781	58.378	40.630	15.6 ± 1.9
C15A	15	112.322	8.191	37.8	16.306407	58.378	41.390	16.7 ± 1.9

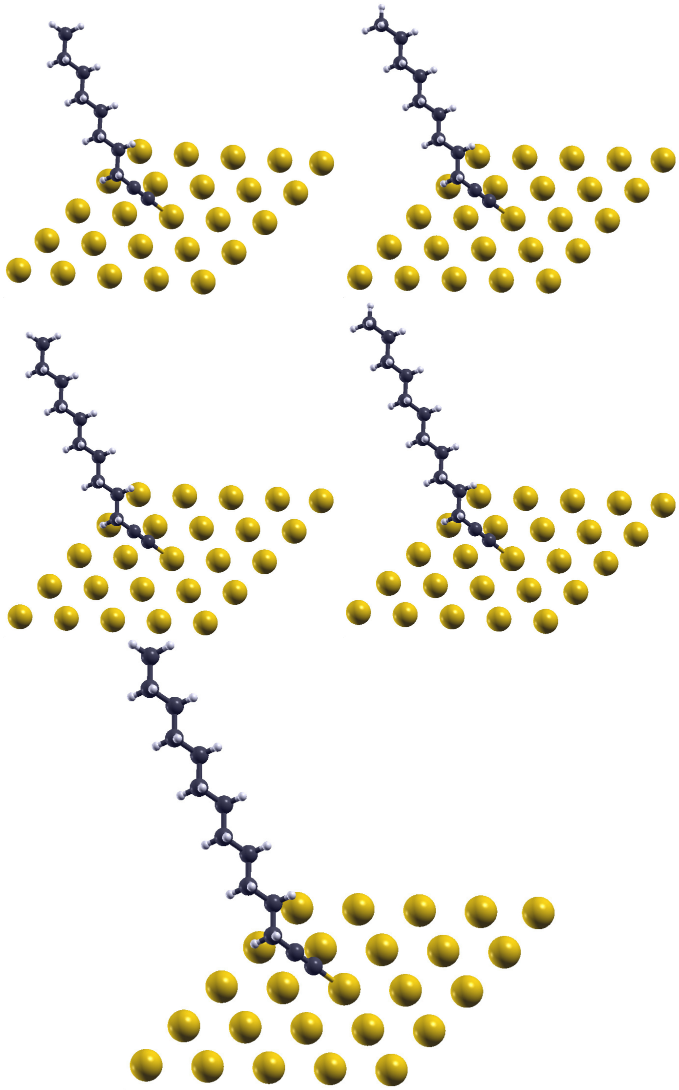

Figure S7: **Structures of "Short" CnA SAMs (n = 8–12).** Orientation and dihedral angle $\alpha \approx 176°$ from dual-group fitting (Section 3.5), closely matching the planar C_s conformer (180°) from gas-phase DFT calculations. Figure generated using XCrySDen [2].

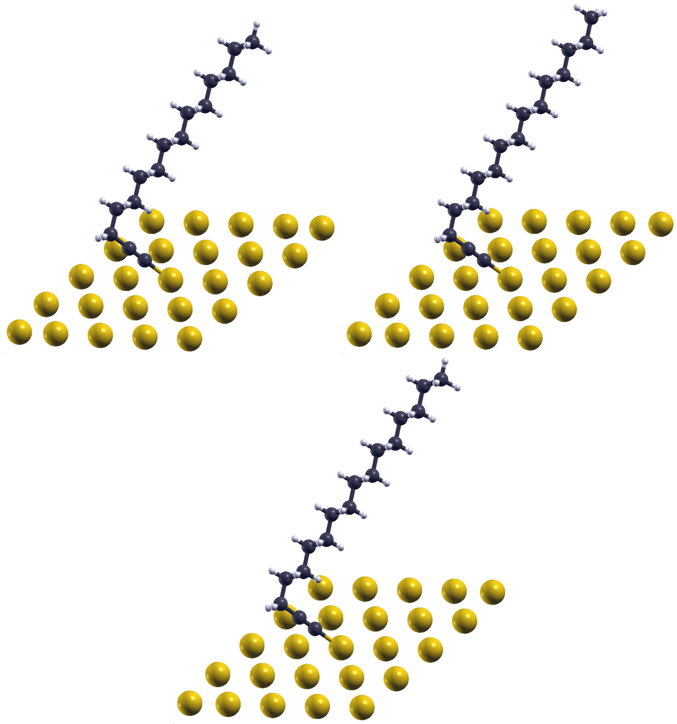

Figure S8: **Structures of "Long" CnA SAMs (n = 13–15).** Orientation and dihedral angle $\alpha \approx 65°$ from dual-group fitting (Section 3.5), matching the skewed C_1 conformer (65°) from gas-phase DFT calculations. Figure generated using XCrySDen [2].

References

[1] M. J. Frisch, G. W. Trucks, H. B. Schlegel, G. E. Scuseria, M. A. Robb, J. R. Cheeseman, G. Scalmani, V. Barone, G. A. Petersson, H. Nakatsuji, X. Li, M. Caricato, A. V. Marenich, J. Bloino, B. G. Janesko, R. Gomperts, B. Mennucci, H. P. Hratchian, J. V. Ortiz, A. F. Izmaylov, J. L. Sonnenberg, D. Williams-Young, F. Ding, F. Lipparini, F. Egidi, J. Goings, B. Peng, A. Petrone, T. Henderson, D. Ranasinghe, V. G. Zakrzewski, J. Gao, N. Rega, G. Zheng, W. Liang, M. Hada, M. Ehara, K. Toyota, R. Fukuda, J. Hasegawa, M. Ishida, T. Nakajima, Y. Honda, O. Kitao, H. Nakai, T. Vreven, K. Throssell, J. J. A. Montgomery, J. E. Peralta, F. Ogliaro, M. J. Bearpark, J. J. Heyd, E. N. Brothers, K. N. Kudin, V. N. Staroverov, T. A. Keith, R. Kobayashi, J. Normand, K. Raghavachari, A. P. Rendell, J. C. Burant, S. S. Iyengar, J. Tomasi, Cossi, M. Millam, Klene, C. Adamo, R. Cammi, J. W. Ochterski, R. L. Martin, K. Morokuma, O. Farkas, J. B. Foresman, D. J. Fox, Gaussian, inc., Wallingford CT, Gaussian 16, Revision b.01, **2016**, URL `www.gaussian.com`.

[2] A. Kokalj, *Comp. Mater. Sci.* **2003**, *28*, 2 155 , proceedings of the Symposium on Software Development for Process and Materials Design.

[3] Y. Chen, I. Bâldea, Y. Yu, Z. Liang, M.-D. Li, E. Koren, Z. Xie, *Langmuir* **2024**, *40* 4410.

[4] I. Bâldea, *Phys. Rev. B* **2012**, *85* 035442.

[5] T. Hastie, R. Tibshirani, J. Friedman, *The Elements of Statistical Learning: Data Mining, Inference, and Prediction*, Springer, New York, NY, 2nd edition, **2009**, second Edition.

[6] C. M. Bishop, *Pattern Recognition and Machine Learning*, Springer, New York, NY, **2006**.

[7] K. P. Murphy, *Machine Learning: A Probabilistic Perspective*, Adaptive Computation and Machine Learning series. MIT Press, Cambridge, MA, **2012**.

[8] A. K. Jain, *Pattern Recognition Letters* **2010**, *31*, 8 651, award winning papers from the 19th International Conference on Pattern Recognition (ICPR).

[9] M. Ester, H.-P. Kriegel, J. Sander, X. Xu, In *Proceedings of the Second International Conference on Knowledge Discovery and Data Mining (KDD '96)*. AAAI Press, Portland, OR, **1996** 226–231.

[10] I. Goodfellow, Y. Bengio, A. Courville, *Deep Learning*, MIT Press, **2016**, `http://www.deeplearningbook.org`.

[11] L. van der Maaten, G. Hinton, *Journal of Machine Learning Research* **2008**, *9*, 86 2579.

[12] P. J. Rousseeuw, *J. Comp. Appl. Math.* **1987**, *20* 53.

[13] L. Hubert, P. Arabie, *Journal of Classification* **1985**, *2*, 1 193.

[14] P. E. Laibinis, C. D. Bain, G. M. Whitesides, *J. Phys. Chem.* **1991**, *95*, 18 7017.

[15] I. Bâldea, *ChemRxiv preprint* **2025**, https://doi:10.26434/chemrxiv–2025–85t1q.
